# The (Re) Production of the Genetically Related Body in Law, Technology and Culture: Mitochondria Replacement Therapy

**DOI:** 10.1007/s10728-016-0329-z

**Published:** 2016-07-25

**Authors:** Danielle Griffiths

**Affiliations:** Institute for Science, Ethics and Innovation, The University of Manchester, 3.383 Stopford Building, Oxford Road, Manchester, M13 9PL UK

**Keywords:** Genetics, Reproductive technologies, Mitochondria replacement therapy, Nuclear family

## Abstract

Advances in medicine in the latter half of the twentieth century have dramatically altered human bodies, expanding choices around what we do with them and how they connect to other bodies. Nowhere is this more so than in the area of reproductive technologies (RTs). Reproductive medicine and the laws surrounding it in the UK have reconfigured traditional boundaries surrounding parenthood and the family. Yet culture and regulation surrounding RTs have combined to try to ensure that while traditional boundaries may be pushed, they are reconstructed in similar ways. This paper looks at the most recent RT to be permitted in the UK, mitochondria (mtDNA) replacement therapy (MRT). Despite controversial media headlines surrounding the technique, MRT is in fact an example of how science and regulation seek to expand models of traditional relatedness in a way that doesn’t challenge the existing order. Yet, like other RTs, while attempts are made to ensure it doesn’t push traditional boundaries too far, fissures and inconsistencies appear in law and culture, which give interesting insights into how genetics, parentage and identity are being mediated in new but familiar ways.

## Introduction

Advances in medicine in the latter half of the twentieth century have dramatically altered human bodies, expanding choices around what we do with them and how they connect to other bodies. Nowhere is this more so than in the area of reproductive technologies (RTs). Traditionally, families were composed of two parents (heterosexual) living with genetically related children conceived within and gestated by the female partner. Rising rates of physical and social infertility^1^ have meant that technology and cultural desires to reproduce have entwined in order to allow many more people to have children in ever more diverse ways. Techniques such as in vitro fertilisation (IVF), surrogacy and donor conception (DC) have changed how bodies reproduce, altered which bodies can reproduce and created new relationships between bodies involved in reproduction. In the United Kingdom, these reproductive advances have benefited from certain legislation, in particular the Human Fertilisation and Embryology Act 1990 (HFE Act 1990) which established the independent regulatory agency the Human Fertilisation and Embryology Authority (HFEA). This has ensured, in most part, robust regulation and the facilitation of medical and scientific progress [25].

Reproductive medicine and the laws surrounding it in the UK have thus reconfigured traditional boundaries surrounding parenthood and the family. There is now no straightforward link between a woman gestating a child, an individual’s gametes and who will eventually become the legal parent(s). This fundamentally alters what relatedness means in the context of reproduction; gestational and genetic ties no longer necessarily make a child’s parent. Of course, there have always been cases where parenthood is divorced from gestation and/or genetics in this way, for example in adoption or partial surrogacy.^2^ Yet in the era of RTs this has become increasingly prevalent, ever more distanced from traditional forms of reproduction and socially acceptable [24]. Technology has thus pushed not only biological boundaries but also legal and cultural ones in the area of relatedness, perhaps further than anyone had ever initially imagined.

Yet legal and cultural boundaries push back. Culture and regulation surrounding RTs have combined to try to ensure that while traditional boundaries may be pushed, they are reconstructed in similar ways. The HFE Act 2008, while transgressive in many ways, has been argued to rely on normative assumptions about the family, in particular the two parent, (hetero)sexual genetically based model of the family [22]. Dean Spade has argued, ‘the assumption that changing what the law says about us will change our lives … misses how the law is often one tactic that rearranges just enough to maintain the current arrangements’ [36]. Yet such maintenance of order is never exact. As science moves on and creates ever-greater possibilities in the realm of reproduction, inconsistencies, fissures and sites of inclusion and exclusion appear both in law and in culture. Brazier has noted that ‘British law displays contradictions, no single, coherent philosophy underpins the law’s response to reproductive medicine’ [7]. The law’s response is often underpinned by prevailing cultural norms, which themselves are often contradictory and shifting, and a failure to reassess what parenthood means in the context of reproductive technologies [7]. This failure results in the contradictory versions of parenthood that are currently recognised in legislation.

This paper will explore such ‘tactics’ to ‘maintain the current arrangements’ [36], and the contradictions and tensions that are produced, through looking at the most recent RT to be permitted in the UK, mitochondria (mtDNA) replacement therapy (MRT). [17] Despite being represented as potentially transgressive [3], MRT is in fact an example of how science and regulation seek to expand models of traditional relatedness in a way that doesn’t challenge the existing order. Yet, like other RTs, while attempts are made to ensure it doesn’t push traditional boundaries too far, fissures and inconsistencies appear in law and culture [24]. This gives interesting insights into how genetics, parentage and identity are being mediated in new but familiar ways.

In order to explore these arguments, I will firstly situate RTs within the context of the idealised form of family relatedness, the hetero-normative family in order to explore how such technologies have challenged but also reinforced this normative family and what tensions and fissures have been produced. I will then look at the development of MRT, their journey through legislation and the ethical issues raised in public, policy and academic debates. The paper will then go on to explore how debates and regulations surrounding this technique say a lot about where we are headed with ‘genetic thinking’ and relatedness, little of which can be described as radical. The fissures being created as part of these regulations will be discussed, including the current inconsistencies in the way that the law attributes parenthood, arbitrarily judging only some genes to matter when defining parenthood and in the seepage of those who cannot fit normalised models of the family. The paper will conclude by reflecting on the future of genetic relatedness, regulation and RTs.

## The Hetero-Normative Genetic Family, Reproductive Technologies and the HFE Act 2008

### The Hetero-Normative Family and Genetic Relatedness

Lauren Berlant used the term hetero-normativity to refer to an idealised family relationship based on a private, intimate and monogamous couple relationship [4]. The most idealised form is a heterosexual couple, joined through a formally celebrated union, living with genetically related offspring. Such a family has been and is deeply ingrained in cultural consciousness, continually normalised and reproduced through cultural and social norms. McCandless and Sheldon [24] show how this genetically related hetero-normative family is also at the centre of family law and legislation. It is assumed to be the way most families *are,* and also a norm for the way family relatedness *shouldbe* [14].

Of course, there are now many diverse forms of family yet only some are legally and culturally validated. The Marriage (Same Sex Couples) Act 2013 and the Civil Partnerships Act 2004 mean that it is not just heterosexual couples who inhabit the intimate and formally recognised norm. Yet regardless of sexuality, hetero-normativity exists to sanction monogamous, sexual, private couple relationships while excluding non-monogamous, polyamorous, public or non-sexual families [4]. Thus while same sex relationships are culturally and legally validated, many within queer studies argue that the fight for civil partnerships and same sex marriage is simply submitting to heterosexual norms and acts to further stigmatise those who don’t inhabit such couple/family sexual relationships [22]. Families where there are more than two people who would like to claim parenthood are not recognised in law or culture.^3^ Non-sexual families are also delegitimised even if there are only two [22].^4^ As McCandless states, in many mooted reforms to family law the conjugal couple remains valorised as the core unit around which families should be recognised and regulated, as opposed to other organising concepts such as care, (inter) dependency, vulnerability or even child welfare. This is despite evidence to show that alternative parenting structures, such as those involving friends or grandmothers and mothers, can potentially improve the welfare of children, parents, families and society [21].^5^

Also central to the heteronormative family is the emphasis on genetic ties between (two) parents and their children. Kinship systems in the West have traditionally been based on the ‘social interpretation of the ‘biological’ fact of reproduction’ [9] and western definitions of kinship refer to genetic categorisation of bodies as means for defining a parent and their child [27, 28]. Such emphasis on genetic relatedness has a long history linked to inheritance, lineage and genealogy. It is a specific symbolic system that is markedly Eurocentric and recent. For example, as many anthropologists have shown, many non-western and historical versions of kinship do not rely on knowledge about physiological procreation or notions of individual paternal consanguinity [29, 37, 38]. Yet while the biogenetic model for defining kin may be relatively recent, modernist, ethnocentric and hetero-normative, it is powerful.

While parenthood is increasingly established through gestational and/or social links, the importance of genetic links between parents and their children has been and is still central to the formation of kinship bonds and identity in Western societies both in culture and law [15, 16]. The cultural discourse of genetics works to “stabilize a narrow and powerful definition of motherhood and fatherhood based on testable biological attributes” [29]. It validates some versions of parenthood (genetic) just as it invalidates others (non genetic) and it has resulted in a specific type of parenthood that is defined through seemingly fixed and law like biological attributes. Chateauneuf and Ouellette [10] state that biomedical explanations for the transmission of genetic links in reproduction create and reinforce notions about family ties having a permanent quality that subsists through time, from one generation to the next. Through science they become the family ties that matter and this resonates deeply in people’s views on parenthood. In social terms, what’s termed “the geneticisation of family relationships” can be considered a more stable way of defining kinship which emphasises continuity in family relationships rather than change [10, 27]. Thus when it comes to decisions over how to have children, preference for having a genetic child dominate over all other forms due to the desire to have strong, lasting ties to one’s children through sharing a common genetic make up [10]. Genetic ties are not only seen to guarantee strong and lasting links they are also often emphasised in relation to being a ‘good’ and ‘proper’ parent. For many, it is assumed that a genetic link will guarantee a ‘better’ parent than a parent who has no genetic link [28]. Appleby and Karnein [3] discuss the wide body of opinion that believes genetic parents are fundamental to a child’s welfare. For example, David Vellman argues that it is vital that we connect our self to our body. Our genetic family helps us to do this as they are ‘in the best position to help us understand who we are’ [40]. The cultural attachment to genetic relatedness is also reflected and normalised in laws surrounding parenthood, albeit in a complex way [11]. Such emphasis on genetic relatedness and the interplay between bodies, culture, law and science can be understood in Foucauldian terms as the process of power that flows between bodies culture and scientific frameworks to a legal system that enacts the resultant technologies to maintain and manage that framework [29].

Such discourses about genetic parents are, of course, easily critiqued. There is much evidence to show that children raised by non-genetic parents do not fare worse and secondly where they do, it usually results from societal attitudes about non-genetic families rather than any biological fact about genetic parents being ‘better’ [19, 34].^6^ In any case, new bodies of scientific opinion are showing that the genes we inherit from our parents are relatively small and those we do share can be changed. We have come to recognise that phenotypes and behavioural patterns are not wholly determined by genetic data but by the interaction of the genetic information with the environment (both internal and external) through a process known as epigenetics [32]. The way we are has always resulted from the complex interplay of our genomic and epigenomic individuality [6, 32]. However the discourse of the genetic parent still rules. As Appleby and Karnein [3] point out, challenging such a discourse, however easy that is, does little to impact on the narrow version of parenthood based on biological attributes. Such forms of parenthood are deeply ingrained in cultural discourse and in legislation. For many parents, the desire to have genetically related children is fundamental [37, 39].

### RTs Fragmenting the Two Parent ‘Genetic Family’?

With the advent of IVF in the 1970s, the development of reproductive technologies have created fissures among these heteronormative forms of the family. The use of donated gametes and/or a third party’s gestational labour means that parenthood and kinship has come to be based on intentional, and/or gestational ties as well as or instead of genetic links. Such techniques reveal that the heteronormative, genetic, sexual, two parent family is socially constructed and just one form among many. It is now possible to have more than two parents who may or may not be in a sexual partnership and who may or may not be genetically related. Furthermore, these non-traditional families are faring very well [19].

The law has in many ways supported such fragmentation and allowed for a flourishing of parental forms which undermine genetic attachment. In cases of reproductive technologies, legal parenthood is determined by the Human Fertilisation and Embryology Act 2008. In cases of donor conception and surrogacy, the birth mother is always the legal mother of a child regardless of whether she is the genetic parent or the intended mother (s.33). In determining fatherhood, a man whose wife or partner has used donated sperm will be treated as the legal father unless consent was not granted by him (s.36-7). Since 2008 two women can be recognised as a legal parent from birth, gestational mother by virtue of birth and her partner by the same provision that allows men to be automatically recognised as fathers where a sperm donor has been used (s.42). In cases of surrogacy the provisions also allow for intending parents to apply for a parental order, which is a fast track form of adoption which extinguishes legal parenthood from the gestational mother/surrogate and grants it to the intending parents (s.54).

Yet science, culture and law in this area have, in other ways, left the genetic family intact. Through the creation, use and regulation of RTs there has not been a collapse of the dominance of the heteronormative family yet neither have they left it intact, they have instead created fissures and more sites of inclusion and exclusion to normalised forms of genetic kinship. Most fundamentally, all ARTs centre on the desire to have genetically related kin. IVF, surrogacy, and even donor conception all centre on the desire to use one’s own or at least one partner’s gametes to produce a child. A lot of money is invested into exploring new ways of allowing people to have genetically related children [24]. Empirical studies with infertile couples have shown that when faced with the news that one or both are infertile and considering options there is a sliding scale of using IVF using one’s own gametes as a first resort, descending to DC with one partner’s gametes, to DC with none of your gametes but gestated by intending mother with adoption of a non genetically related child at the very bottom [10, 39]. Many academic discussions of RTs do not interrogate this desire for genetically related children and when they do they simply assert its importance to people. For example, discussion of alternatives to RTs usually conclude that parents ‘may feel that it is important to have a genetic link with their future child and that having this genetic link outweighs most disadvantages’ [2]. Such lack of interrogation does not challenge the cultural dominance of genetic links.

While the law has undermined the heternormative family in some ways, in others it has maintained and strengthened them. The 2008 HFE Act allows in some ways for the creation of non-genetic families, yet it has left undisturbed one of the key assumptions underlying the heteronormative family, the two parent, sexual family [23]. The Department of Health did discuss early on the possibility of allowing more than two parents to be recognised but it was explicitly rejected on the basis that the consequences of this would be too radical and controversial [23]. The Act never considered whether having more than two parents might benefit children [21]. It denies people who are in non-sexual partnerships the right to apply for legal parenthood [22]. In surrogacy arrangements it is also a condition of a parental order that at least one of the intending parents has a genetic connection to the child, thus again asserting the importance of genetic links [1]. Single people are also not allowed to apply for a parental order.

The continued dominance of the heteronormative family and the tensions that RTs have brought in law and culture is revealed most starkly in debates around donor-conceived children. Donor anonymity was removed for gamete donors in April 2005 following the HFEA (Disclosure of Donor Information) Regulations 2004. It is not compulsory to tell but recommended [30]. Underlying and accompanying this is the disocurse of genetic relatedness which asserts that knowledge of genetic origins is fundamental to donor-conceived children [35]. Thus while the law supports the proliferation of non-genetic families through its parental provisions, it also reaffirms the importance of genetic ties through the removal of donor anonymity. McCandless [22] has argued that donor anonymity in the past was a key method of shoring up the two-parent, genetic family. It allowed people to ‘pass’ as genetic relations and/or not be threatened with the third party donor destroying the illusion of heternormativity. Removal of anonymity is a result of strengthening discourses related to ‘genetic identity’ but also because of an increased confidence in the security of parental ties formed by law [22]. Revealing one’s donors is no longer thought to threaten the security of the two-parent nuclear family. Thus there are a number of contradictory discourses at work here in ensuring a RT does not threaten the normative family. Valorisation of genetic ties is central to the law’s shoring up of the heteronormative family but these ties can also threaten this institution.

How such legislation plays out in academic and cultural practice is producing tensions and fissures all of which reproduce rather than undermine the genetic family. On one side of the academic and policy debate for a child’s right to know is that genealogical knowledge is central to the development of personal identity. Here genetic determinism is clear. Nuffield Council recently recommended that it will usually be better for children to be told, by their parents and at an early age, that they are donor-conceived [30]. Others are pushing to go further and make it mandatory for donor conceived people to be told, by, for example, including it on birth certificates [5]. Responding to people’s desires to ‘not know’, the other side of the argument also has strong echoes of genetic determinism. Ravelingien and Pennings [33] point to the harm of denying parents the opportunity to determine how, when or even whether to reveal that they are (both) not genetically related to their children. In particular, they contend that forced disclosure of the use of third party gametes denies parents and families the choice of passing as ‘normal’. ‘The current imperative to be open about the donor denies parents the possibility to maintain a normal appearance in these respects and makes it difficult for the social parent to override the genetic link between the donor and the child’ [33]. Ravelingien and Pennings are affirming that having to maintain secrecy in the context of gamete donation comes from the mandate that proper families are made through biogenetic reproduction [33]. They reveal that the performance of genetic relatedness is mandatory for a family to be recognised as legitimate. Here secrecy not based on a rejection of genetic thinking as if it doesn’t matter but rests on the fact that genes do matter and knowing one’s true progenitor may invalidate other non-genetic relationships. This recognises that many parents don’t feel like they have a choice over telling or not, they feel forced to keep it a secret.

For some, the fact that RTs do produce tensions gives rise to optimism in that they may affirm but they also have the potential to undo genetic attachments in practice [3]. Evidence for the latter however is lacking. Despite being encouraged to tell, most parents do not, if they can avoid it [35].^7^ Those that do are cautious, knowing that it was not just mere information but is ‘powerful knowledge that changes relationships’ [34, 35]. ‘Knowing that one’s niece is not the child of one’s brother has profound effects, it is not just a piece of factual information’. Knowledge of genetic ties or lack of them often overrides and changes existing relationships. Thus RTs have not so much undone genetic connections, a lack of such connection through RTs reasserts the importance of it to many people. Such reassertion has led to increased amounts of secrecy within families who can pass as a genetic, heteronormative family. This then creates new divisions between those that can be normalised and those that can’t, those who can pass and those who can’t.

## Mitochondria Replacement Therapy: Undoing Heteronormativity?

Up until recently regardless of the flourishing of RTs, it was only possible to inherit the genes of two people involved in conception. Techniques of MRT challenge this and present a new challenge to the cultural and legal logic of the heteronormative, genetic two parent ideal.

### Development of the Technique

MtDNA is present in almost all human cells and generate the majority of a cell’s energy. They carry a genome which is distinct from the nuclear genome, their actions are thought to be restricted to governing the actions of the mitochondria only and they make up 0.1 % of our genes in total [31]. MtDNA is maternally inherited. Unhealthy mitochondria can cause genetic disorders known as mitochondrial disease. It is estimated that about one in 200 children born in the UK have some form of mitochondrial disorder, most of these cases are due to a very low mutation and cause only mild forms of mitochondrial disorders or are asymptomatic [31]. However, these mutations could nonetheless be passed on to future children at more significant levels. There are many conditions linked to mitochondria disease, ranging from mild to severe or life threatening. There is no cure for mitochondrial disease and treatment options are severely limited. This means that some people who inherit the condition may live with debilitating illness resulting in the death at an early age of the child who inherits it. Currently women with such condition who wish to have genetically related children without passing on this disorder can use other techniques such as pre-implantation genetic diagnosis and pre-natal diagnosis all of which have their drawbacks [31].

Two techniques termed pro-nuclear transfer and maternal spindle transfer have been developed in order to allow women with mitochondria disease to have genetically related children without passing on the disorder. In the first technique, two eggs are fertilised with sperm, creating an embryo from the intended parents and another from the donor egg. The pronuclei, which contains genetic information, are removed from both embryos. The pronuclei from the intending parents is inserted into the enucleated embryo which carries the healthy donor mitochondria [2]. In the second technique eggs from a mother with damaged mitochondria and a donor with healthy mitochondria are collected, the nucleus is removed from both eggs, the mother’s nucleus is inserted into the donor egg, which can then be fertilised by sperm. As a result of both, a baby will have DNA from the biological parents and a female donor who provides healthy mitochondria [2].

Numerous consultations were launched which explored key safety and ethical issues [12, 18, 31]. Most policy and academic concern centred on the safety of the technique, the impact of a child having DNA from three people, whether it constituted a form of germline modification and if so the implications of it, and whether allowing the technique would lead to a slippery slope to other less acceptable things [38]. In much debate and the recommendations resulting from these concerns was wide agreement that this is a technique that will prevent suffering in future persons and most ethical issues were not serious enough to warrant banning the technique. In December 2014 the government published the Human Fertilisation and Embryology (Mitochondrial Donation) Regulations 2015.^8^ In early 2015 the House of Lords peers vote by 280 to 48 in support of the Regulations^9^ and in the House of Commons MPs vote by 382 to 128 to pass them.^10^ The Regulations came into effect in October 2015. The UK became the first and only country in the world to legalise this germ-line technology and has been celebrated for being at the ‘vanguard of mitochondria science’ [13].

### MRT: ‘Rearranging Just Enough to Maintain the Current Arrangements’

While much previous work has heralded its radical potential, less attention has been paid to how this new RT is reproducing the existing order in new but familiar ways. Some have argued that MRT has the potential to undermine the traditional nuclear family as it leads to the creation of children with contributions from three people [3]. The traditional genetic family potentially cannot contain its two parent boundaries and thus MRT could produce changes to the heteronormative ideal. Yet the creation of MRT and the regulations surrounding it show a more conservative picture, one that again reproduces the heteronormative family.

Firstly, genetic relatedness is the impulse behind mtDNA replacement. The techniques will certainly not eradicate mitochondria disease and will not significantly reduce numbers being born with it. The whole impetus is to allow these women to conceive a healthy child with their own genes. As stated earlier, this desire is often accepted uncritically. For example, Brendenoord states, ‘as having healthy, genetically related children is for many people one of the most important desires in life, helping couples to fulfil this desire is a legitimate aim of a reproductive technique such as nuclear transfer for mtDNA disease’ [8]. While recognising this desire for genetically related children may be poor use of resources, Stephen Wilkinson states the argument that people should use adoption or egg donation instead could be used against most RTs including IVF and even gynaecological surgery to cure infertility [41]. Yet distinctions can be made. IVF benefits a huge amount more people than MRT will and gynaecological surgery restores ‘natural’ fertility rather than intervenes in conception to the extent MRT does [31]. These points do not lessen the importance of MRT in order to allow women with faulty MtDNA to have genetically related children, nor does it mean that MRT should be banned and resources directed elsewhere. It is simply to state that having a child with your own genes remains deeply embedded in culture, science and law and when it comes to assisting even just a handful of women to have children related to them, then the resources and effort required are subsumed beneath the imperative to fulfil this desire.

The danger of such an imperative to have genetically related children, whatever RT is used, is that some parental relationships are validated and normalised while others are not. As genetic relatedness becomes ever more available for some through RTs, and as its importance is recognised and given precedence in the science and laws that make RTs possible, then tensions are produced in distancing those who can’t pass as a genetic family ever more from the heteronormative ideal. If science, culture and law did not shore up the genetic ideal and if then parenthood were to become more fluid and based on numerous other ties aside from genetics then maybe using an egg donor or adoption may be a more desirable and easier option than this complex technique. Such fluidity would result in less exclusion from the heteronormative ideal.

Secondly this emphasis genetic relatedness only stretches so far, only some genes count when it comes to the genetic ideal. As argued earlier and seen in relation to DC and surrogacy, the law’s response to reproductive technologies is often underpinned by prevailing cultural norms rather than an effort to reassess what parenthood means [7]. This is the case in the formulation of regulation surrounding MRT. Much consideration in the debate on MRT was given to the status of the mitochondria donor. Namely should the MtDNA donor be treated the same as a gamete donors with the children born through MRT having a right to access identifying information about the donor when they reach the age of 18 or should the donor be anonymous and have a similar status to tissue donors? That is, consultations were asking which genes matter and how many when it comes to parentage and genetic heritage. In most of the reports and recommendations produced, the significance of MtDNA to a person’s identity was played down. It was continually emphasised that MtDNA contains only 0.1 % of the total amount of DNA and that it does not alter essential characteristics or identity determining characteristics, it is the power house of a cell and nothing else. In the Human Fertilisation and Embryology (Mitochondrial Donation) Regulations 2015 ‘the applicant may request the Authority to give the applicant notice stating whether or not the information contained in the register shows that a person is the applicant’s mitochondrial donor’ but ‘not giving any information which may identify the mitochondrial donor’. Such anonymity of the MtDNA donor is starkly at odds with regulation surrounding DC.

Is it so straightforward to write out the MtDNA donor from identity and parentage? Much of the policy reports and media reporting present very simplistic accounts of genetics ‘genes for’ characteristics such as eye colour which does not recognise the complexity of genomics [20]. The significance of MtDNA has been contested. Bredenoord et al. argues that it is not a fully settled issue in science that only nuclear DNA contains the ingredients for our characteristics [8]. Some studies suggest associations between mtDNA and cognitive capabilities. Caroline Jones and Ingrid Holme also highlight the role mtDNA plays in identification, MtDNA is a validated technique for the identification of skeletons in forensics and can be used in genealogy [20]. It has also been used for ‘ethnicity testing’ for children with unknown parentage in the care system. MtDNA clearly has some effect on identity in these circumstances. Also the donor’s DNA affects the recipient’s identity in the most fundamental way, that of health status, without the healthy MtDNA the child’s identity would be potentially characterised by disease [8]. While MtDNA does clearly have some contributions to our identity, it is true that it does not have the same significance as nuclear DNA. But should quantity matter? When we are so gripped by the importance of genetic links then it seems a contradiction for MtDNA to not matter. If a donor conceived person is given the right to access information on their donor due to the view that knowing their genetic heritage is good for their psychological wellbeing, is it wrong to assume that a person born through MRT will not benefit in the same way. Wilkinson asks the same question. ‘There are already lots of families that only exist because of the biological input of a third person: for example, ones created using traditional egg donation, or surrogacy… it’s not clear that “three parent IVF” is all that different from practices that we already accept’ [41]. Regulations surrounding MRT seem to be another example of the law’s contradictory and shifting response to RTs, which is most often underpinned by prevailing cultural norms.

In a HFEA public meeting on MRT in March 2013, discussion focused on the ‘three parent issue. The reply by many of the members was that if donors were not to be anonymous and were in any way treated as a genetic parent, it played too much into the three-parent issue, which had proved most controversial in media coverage. Many viewed that recommending the ‘third parent’ as identifiable could jeopardise the whole technique getting through parliament. The empirical research they conducted supported this [18]. Most respondents saw the donor as having some connection but expressed concern over treating them the same as a gamete donor as they thought the child’s identity may suffer negatively from the social effects of having the three-parent tag. Making the donor’s identity not matter in regulations reveals a lot about how much it matters.

Yet it still remains to be explained why ties formed by the law in the case of DC are deemed to be so secure that identification of a donor would not jeopardise the two parent hetero-normative family [22] but not in the context of MRT. Perhaps Appleby and Karnein [3] were actually correct to argue MRT could undermine the nuclear family as for the first time three people will be contributing to the genetic make up of a person. In DC there is a genetic contribution from just two people and it follows the model of adoption where knowledge of the genetic parents has not in most part undermined the relationship with adoptive parents thus the law is less uneasy, even if many parents are more uneasy and continue not to tell. Yet despite the law pushing the donor to the outskirts, in MRT there may be less chance of passing as a two parent genetic family as there is in DC as the person born through MRT will be encouraged to have follow-up consultations [41]. McCandless and Sheldon point out the law may say one thing but it remains to be seen how the children born through MRT will see it if and when they do find out [24]. Yet as they note, a significant part of that reaction will be shaped by how the law interprets it. As the law undermines this genetic link while it valorises others, the hetero-normative family may be protected but in a contradictory and incoherent way.

## Conclusion

Reproductive medicine and regulation surrounding it has certainly reconfigured some traditional boundaries surrounding parenthood and the family. They promote certain choices in how to organise one’s family and make family making possible for many who were unable to. Yet the potential of these technologies to radically undermine the hetero-normative family in order to allow space for alternative forms is limited. Most RTs have as their basis the desire for genetically related kin. Regulation of them has also been conservative. McCandless and Sheldon [24] note, there is no ‘objective, scientific base on which we can hope to construct a perfect regulatory edifice’ in the context of RTs. Relatedness is socially constructed and defined according to shifting cultural and legal norms. In regulating mtDNA, like with previous RTs, legislation has again sought to order and re-centre the hetero-normative family. Does this matter? In so far as it validates and invalidates different kinds of parental relationships and generates a huge amount more secrecy within family relationships then it perhaps does matter.

Technologies on the horizon such as artificial gametes also have as their fundamental aim to enable people to have genetically related children [32]. This technology also promises wider opportunities such as allowing both members of same sex couples to engage in genetic parenting, allowing just one or more than two persons to engage simultaneously in genetic parenting. While this would certainly challenge the two-parent focus of the genetic family, it is hard to see that the law would ever allow this when in MRT it was deemed too disruptive of the two-parent ideal to identify MtDNA donors. Until we challenge the contradictory processes by which relatedness is determined in law and culture, RTs and the science behind them are not going to radically alter the choices around what we do with our bodies and how we’re connected to other bodies. In order to make all kinds of relationships valid and reduce secrecy we need to challenge the larger processes which determine what counts as family. The law forms a significant part of this as it gives cultural validation and recognition. If the normalisation of two parent genetic kinship is undermined through law then it may matter less how many genetic parents we have or whether they are genetically related at all.

## Footnotes

1 It is estimated that 1 in 7 women will experience problems concieving [26]. Social infertility refers to groupswhocannotreporduce for reasons related to sexuality or relationship status e.g. gay couples and single people.

2 Where a surrogate uses her own egg and sperm from the intended father. The intended mother therefore has no genetic or gestational relationship to the child. Actual incidence of this is unknown but is said to date back to biblical times. Using an egg from the intended mother (full surrogacy) only became possible with the widespread use of IVF in the 1980s.

3 They could be in a sexual relationship (e.g. polymorous relationships) or not (e.g.in donor conception families where the donors play an active parenting role in the donor child’s life.

4 For a recent case of a couple who are seeking to challenge this exclusion of non-sexual families being recongised in law and which indicates the importance of legal recongition for things such as inheritance rights and tax see http://www.theguardian.com/lifeandstyle/2016/jan/23/alternative-family-law-inheritance-tax-money?CMP=share_btn_fb. For an older case involving two sisters who lived together as a family all of their lives and who sought the same ininheritance tax rights to that of a married couple which got to the European court of human rights in 2008, see http://www.theguardian.com/money/2008/apr/29/inheritancetax.humanrights.

5 M. Lotz, ‘The Two-Parent Limitation in ART Parentage Law: Old-Fashioned Law for New Fashioned Families’, in in D. Cutas and S. Chan (eds) *Families Beyond the Nuclear Ideal,* Bloomsbury 2012.

6 This is particularly so in relation to donor concieved families where at least one parent is not genetically related [23].

7 Hiding the fact a donor was used is of course impossible in some families such as same sex couples..

8 http://www.legislation.gov.uk/ukdsi/2015/9780111125816/contents.

9 http://www.parliament.uk/business/news/2015/february/lords-mitochondrial-donation-si/.

10 http://www.parliament.uk/business/news/2015/february/commons-debate-statutory-instrument-on-mitochondrial-donation/.
